# MiR-16-5p regulates postmenopausal osteoporosis by directly targeting VEGFA

**DOI:** 10.18632/aging.103223

**Published:** 2020-05-19

**Authors:** Tao Yu, Xiaomeng You, Haichao Zhou, Wenbao He, Zihua Li, Bing Li, Jiang Xia, Hui Zhu, Youguang Zhao, Guangrong Yu, Yuan Xiong, Yunfeng Yang

**Affiliations:** 1 Department of Orthopedic Surgery, Tongji Hospital, Tongji University School of Medicine, Shanghai 200065, China; 2 Department of Orthopedic Surgery, Brigham and Women’s Hospital, Harvard Medical School, Boston, MA 02115, USA; 3 Department of Orthopedics, Union Hospital, Tongji Medical College, Huazhong University of Science and Technology, Wuhan 430022, China

**Keywords:** osteoporosis, osteogenesis, bone mass density, miR-16-5p, VEGFA

## Abstract

In this study, we used bioinformatics tools, and experiments with patient tissues and human mesenchymal stem cells (hMSCs) to identify differentially regulated genes (DEGs) and microRNAs (miRNAs) that promote postmenopausal osteoporosis. By analyzing the GSE56815 dataset from the NCBI GEO database, we identified 638 DEGs, including 371 upregulated and 267 downregulated genes, in postmenopausal women with low bone density. Enrichment and protein-protein interaction network analyses showed that *TP53, RPS27A, and VEGFA* were the top three hub genes with the highest degree of betweenness and closeness centrality. TargetScanHuman and DIANA software analyses and dual luciferase reporter assays confirmed that miR-16a-5p directly targets the 3’UTR of VEGFA. Postmenopausal patients with osteoporosis showed higher miR-16-5p and lower VEGFA levels than those without osteoporosis (n=10 each). VEGFA levels were higher in miR-16-5p knockdown hMSCs and were reduced in miR-16-5p-overexpressing hMSCs. mRNA expression of osteogenic markers, ALP, OCN, and RUNX2, as well as calcium deposition based on Alizarin red staining, correlated inversely with miR-16-5p levels and correlated positively with VEGFA levels. These findings suggest that miR-16-5p suppresses osteogenesis by inhibiting VEGFA expression and is a promising target for postmenopausal osteoporosis therapy.

## INTRODUCTION

Osteoporosis is characterized by reduced bone mass and weakened bone micro-architecture, resulting in an increased risk of fractures [1]. Osteoporosis is a common age-related disease in post-menopausal women and the elderly [2]. It affects the quality of life of nearly 200 million people worldwide and is a significant burden on the public healthcare systems [3]. Nearly 40% of women suffer from osteoporosis and sustain fractures of the hip, spine, or the forearm during their lifetime [4]. While there are several medications to treat osteoporosis based on their symptoms and severity, there is no gold standard treatment established, as yet [4]. Therefore, it is important to understand the genetic mechanisms underlying osteoporosis so that new therapeutic targets can be identified [5]. Recent studies have identified SQRDL and PPWD1 genes as risk factors that are associated with osteoporosis [6, 7].

miRNAs are small noncoding RNAs between 21–25 nucleotides in length that regulate protein expression through post-transcriptional gene silencing [8–10]. Previous studies have identified differentially expressed miRNAs in osteoporosis patients, including miR-21, miR-133a, miR-152-3p, miR-30e-5p, miR-140-5p, miR-324-3p, miR-19b-3p, miR-335-5p, miR-19a-3p, miR-550a-3p, and miR-422a [11–13]. Moreover, several signaling pathways that are involved in osteoporosis are regulated by microRNAs such as miR-133a, miR-218, miR-618, miR-27a, miR-214-5p, and miR-203 [14–18]. However, the mechanistic details of how these miRNAs influence osteoporosis are not known.

Bioinformatics is an interdisciplinary field that combines computer science and biology to research, analyze and interpret large sets of biological data. Computational tools are routinely used to model biological processes, predict disease mechanisms and generate experimental hypotheses [19]. Bioinformatics tools are also widely used to screen potential genes related to several human diseases, which are then validated by comprehensive follow-up experimental studies [20, 21].

In this study, we analyzed microarray data from the GSE56815 dataset to identify DEGs that are related to osteoporosis. Furthermore, we performed enrichment analysis and constructed an interaction network of the DEGs to identify hub genes. Furthermore, we used the TargetScanHuman and DIANA software to identify microRNAs that potentially target the hub genes and validated these findings in patient tissues and *in vitro* experiments using human mesenchymal stem cells.

## RESULTS

### DEGs in postmenopausal osteoporosis

**Figure 1:**
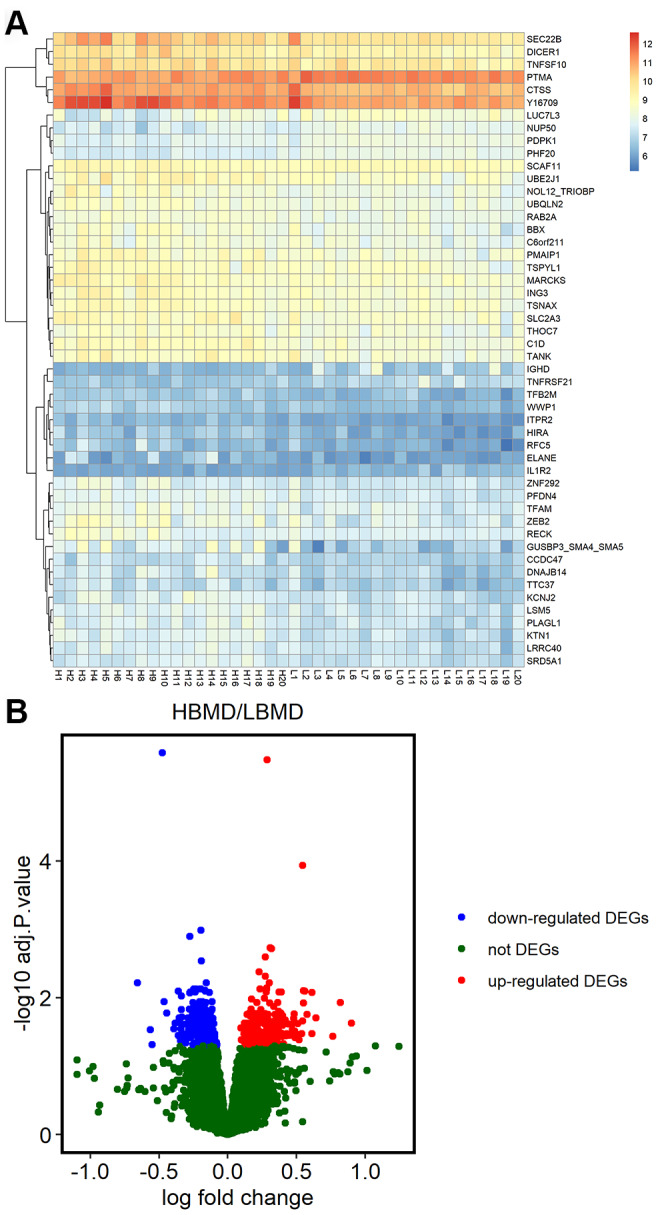
**Differentially expressed genes in postmenopausal osteoporosis patient samples.** (**A**) The heat map shows hierarchical clustering of differentially expressed gene expression in postmenopausal patients with or without osteoporosis (n=20 each) using the GSE56815 dataset. (**B**) Volcano plot shows differentially expressed genes postmenopausal patients with or without osteoporosis (n=20 each) using the GSE56815 dataset.

We analyzed the microarray data from the GSE56815 dataset in the NCBI GEO database, which includes gene expression data on the Affymetrix Human Genome U133A Array (GPL96) platform of 20 postmenopausal women each with high or low bone mineral density (BMD). We identified 638 DEGs, including 371 upregulated and 267 downregulated genes in patients with low BMD compared to those with high BMD (Figure 1B). The heat map showing hierarchical clustering of all DEGs and the volcano plot of the DEGs are shown in Figure 1.

### Construction of the protein-protein interaction (PPI) network of the DEGs

**Figure 2:**
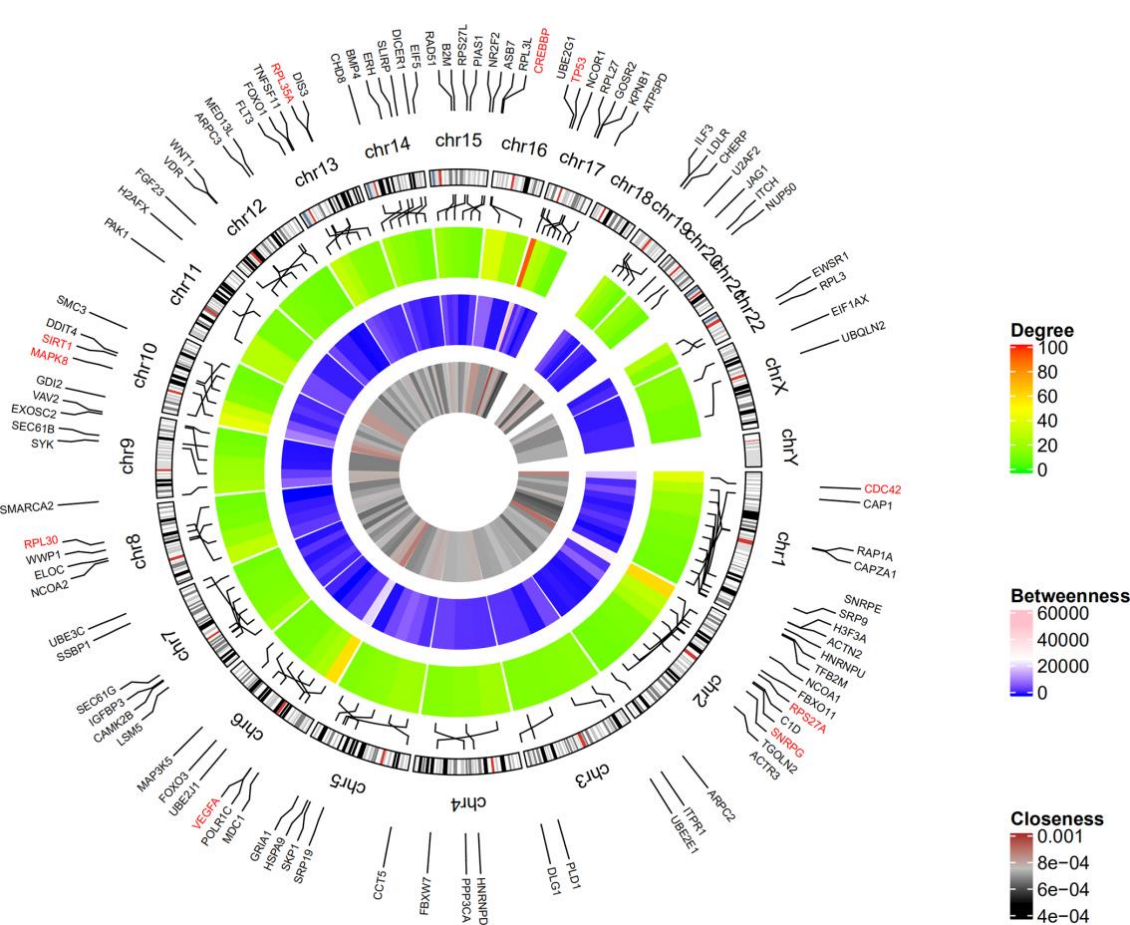
**Circular visualization of chromosomal positions and connectivity of the top 100 genes.** The circular map represents all the chromosomes and the lines from each gene point to their specific chromosomal locations. The names of the DEGs are shown in the outer circle and the three hub genes are shown in red. The red and green colors in the outer heatmap represent DEGs with high and low degree, respectively. The pink and blue colors in the middle heatmap represent DEGs with high and low betweenness, respectively. The brown and black colors in the inner most heatmap represent DEGs with high and low closeness, respectively.

**Figure 3:**
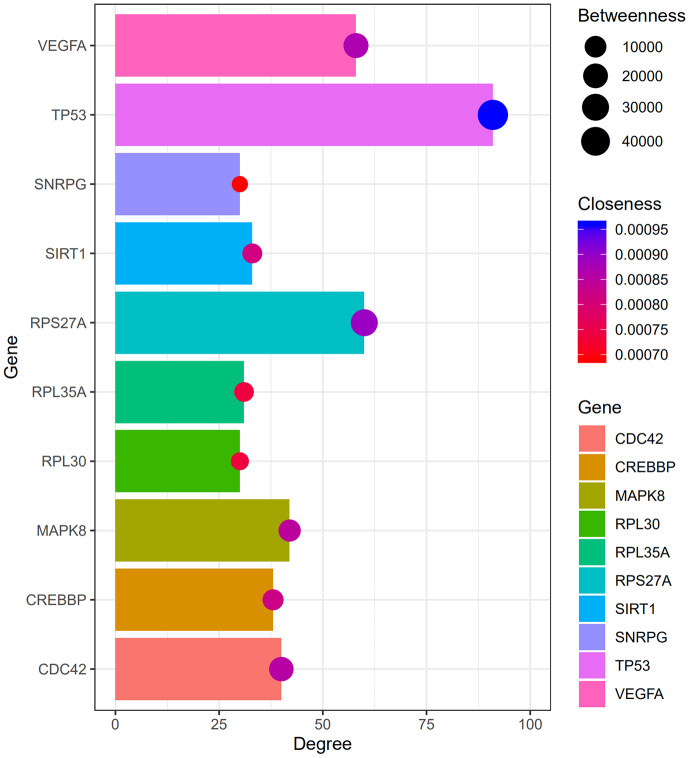
**The degree, betweenness, and closeness centrality of hub genes.** The analysis shows that *TP53, RPS27A, and VEGFA* were the top three hub genes with greatest degree, betweenness and closeness.

Figure 2 shows the top 100 DEGs from the 3 modules and their chromosomal positions. The top 10 hub genes were *TP53, RPS27A, VEGFA, MAPK8, CDC42, CREBBP, SIRT1, RPL35A, RPL30*, and *SNRPG* (Table 1). Figure 3 shows the degree, betweenness, and closeness centrality of the top 10 hub genes. The top three hub genes with the highest degree, betweenness and closeness centrality are *TP53, RPS27A, and VEGFA*.

**Table 1:** Degree of the top 10 genes in the protein-protein interaction network.

**Gene ID**	**Gene name**	**Degree**
*TP53*	Tumor protein p53	91
*RPS27A*	Ribosomal Protein S27a	60
*VEGFA*	Vascular Endothelial Growth Factor A	58
*MAPK8*	Mitogen-Activated Protein Kinase 8	42
*CDC42*	Cell Division Cycle 42	40
*CREBBP*	CREB binding protein	38
*SIRT1*	Sirtuin 1	33
*RPL35A*	Ribosomal Protein L35a	31
*RPL30*	Ribosomal Protein L30	30
*SNRPG*	Small Nuclear Ribonucleoprotein Polypeptide G	30

### Gene ontology (GO) and Kyoto Encyclopedia of Genes and Genomes (KEGG) pathway enrichment analysis

**Figure 4:**
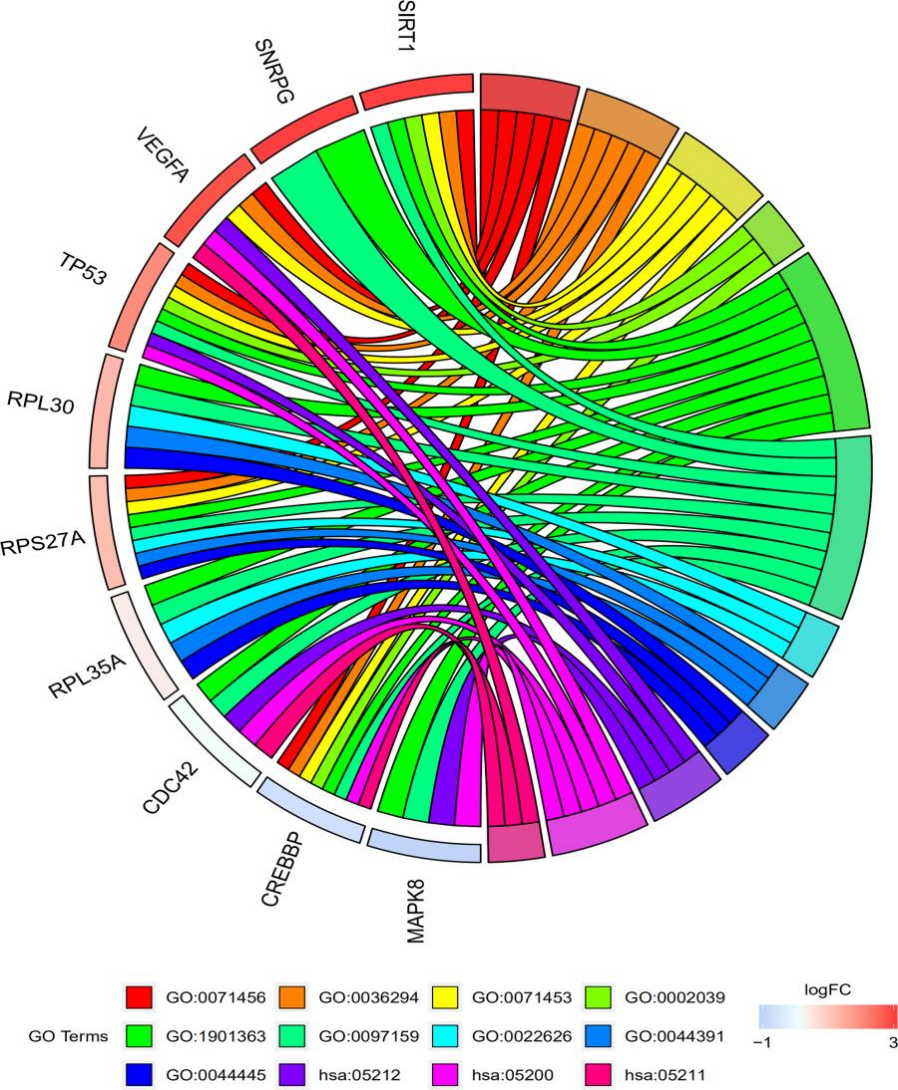
**Functional and signaling pathway enrichment analyses of the top 10 genes in the protein-protein interaction network.** The GO and KEGG pathway analysis of the top 10 genes identified by the PPI network are shown. The top 3 GO terms related to biological process are cellular response to hypoxia (GO:0071456), cellular response to decreased oxygen levels (GO:0036294), and cellular response to oxygen levels (GO:0071453). The top 3 GO terms related to molecular functions are P53 binding (GO:0002039), heterocyclic compound binding (GO:1901363), and Organic cyclic compound binding (GO:0097159). The top 3 GO terms related to cellular component are cytosolic ribosome (GO:0022626), ribosomal subunit (GO:0044391), and cytosolic part (GO:0044445). The top 3 KEGG pathways are pancreatic cancer (hsa:05212), pathways in cancer (hsa:05200), and renal cell carcinoma (hsa:05211).

Table 2 and Figure 4 shows the results of the GO and KEGG pathway enrichment analysis of the hub genes and DEGs using Database for Annotation, Visualization and Integrated Discovery version (DAVID). The top 3 GO terms related to biological processes were cellular response to hypoxia, cellular response to decreased oxygen levels, and cellular response to oxygen levels. The top 3 GO terms related to molecular functions were P53 binding, heterocyclic compound binding, and organic cyclic compound binding. The top 3 GO terms related to cellular components were cytosolic ribosome, ribosomal subunit, and cytosolic part. The top three KEGG pathways were pancreatic cancer pathway, pathways in cancer, and renal cell carcinoma pathway.

**Table 2:** Top 3 GO terms according to biological process, molecular function, and cellular component, and top 3 KEGG pathways related to the top 10 hub genes in module.

**(A) Biological processes**				
**Term**	**Name**	**Count**	**P-value**	**Genes**
GO:0071456	Cellular response to hypoxia	5	2.6E-7	VEGFA, CREBBP, TP53, SIRT1, RPS27A
GO:0036294	Cellular response to decreased oxygen levels	5	3.2E-7	VEGFA, CREBBP, TP53, SIRT1, RPS27A
GO:0071453	Cellular response to oxygen levels	5	4.2E-7	VEGFA, CREBBP, TP53, SIRT1, RPS27A
**(B) Molecular functions**				
**Term**	**Name**	**Count**	**P-value**	**Genes**
GO:0002039	P53 binding	3	6.5E-4	CREBBP, TP53, SIRT1
GO:1901363	Heterocyclic compound binding	9	2.9E-3	CDC42, RPL35A, RPL30, CREBBP, TP53, MAPK8, SIRT1, RPS27A, SNRPG
GO:0097159	Organic cyclic compound binding	9	3.2E-3	CDC42, RPL35A, RPL30, CREBBP, TP53, MAPK8, SIRT1, RPS27A, SNRPG
**(C) Cellular component**				
**Term**	**Name**	**Count**	**P-value**	**Genes**
GO:0022626	Cytosolic ribosome	3	2.0E-3	RPL35A, RPL30, RPS27A
GO:0044391	Ribosomal subunit	3	4.5E-3	RPL35A, RPL30, RPS27A
GO:0044445	Cytosolic part	3	7.1E-3	RPL35A, RPL30, RPS27A
**(D) KEGG pathway**				
**Term**	**Name**	**Count**	**P-value**	**Genes**
hsa05212	Pancreatic cancer	4	4.4E-5	CDC42, VEGFA, TP53, MAPK8
Hsa05200	Pathways in cancer	5	6.1E-4	CDC42, VEGFA, CREBBP, TP53, MAPK8
hsa05211	Renal cell carcinoma	3	2.4E-3	CDC42, VEGFA, CREBBP

KEGG, Kyoto Encyclopedia of Genes and Genomes; Top 3 terms were selected according to P-value.

### Construction of the network of hub genes and related microRNAs

**Figure 5:**
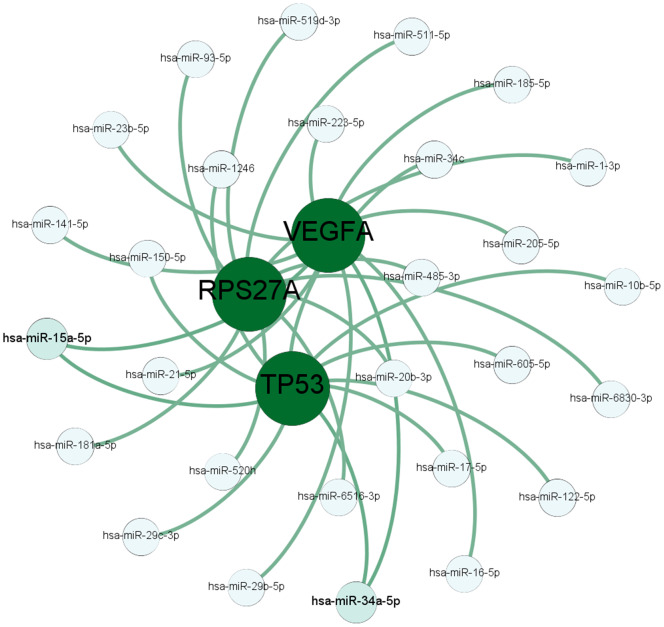
**The miRNA-mRNA targeting prediction network.** The results of TargetScanHuman and DIANA software analyses show the miRNAs that potentially target the mRNAs of the top 3 hub genes, *TP53, RPS27A, and VEGFA*.

Next, we used TargetScanHuman [22] and DIANA [23] software to identify miRNAs that target the hub genes. As shown in Figure 5, hsa-miR-34a-5p potentially targets TP53 and VEGFA, and hsa-miR-15a-5p potentially targets TP53 and RPS27A. VEGFA is highly expressed in osteoblast precursors [24] and induces osteogenic differentiation [25, 26], which suggests its potential role in bone turnover. Previous reports suggest that miR-16-5p, miR-20b-3p, miR-15a-5p, and miR-34a-5p regulate osteogenesis [27–30]. QRT-PCR analysis of serum samples showed significantly high miR-16-5p levels and significantly reduced VEGFA levels in the osteoporosis patients compared with the healthy subjects (Supplementary Figure 1 and Figure 7A). Therefore, we selected the miR-16-5p/VEGFA axis to validate further.

### MiR-16-5p is upregulated in osteoporosis patients and exerts negative effect on osteogenic differentiation

**Figure 6:**
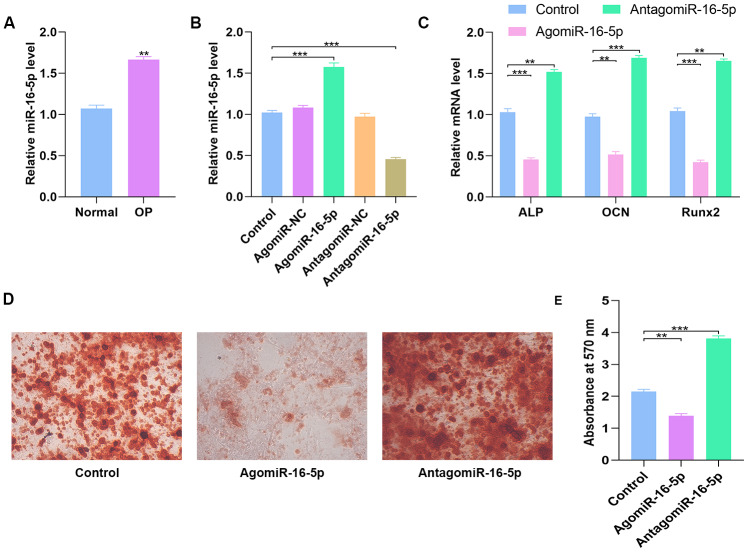
**High miR-16-5p levels inhibit osteogenic differentiation in osteoporosis patients and hMSCs.** (**A**) QRT-PCR analysis of miR-16-5p levels in postmenopausal patients with or without osteoporosis (n=10 per group). (**B**) QRT-PCR analysis of miR-16-5p levels in control hMSCs and hMSCs transfected with agomiR-NC, agomiR-16-5p, antagomiR-NC, and antagomiR-16-5p is shown. (**C**) QRT-PCR analysis shows the relative expression of osteogenic marker genes, ALP, OCN and RUNX2, in control hMSCs and hMSCs transfected with agomiR-NC, agomiR-16-5p, antagomiR-NC, and antagomiR-16-5p. (**D**, **E**) Alizarin red staining shows calcium deposition in control hMSCs and hMSCs transfected with agomiR-NC, agomiR-16-5p, antagomiR-NC, and antagomiR-16-5p for 21 days. Scale bar = 10 mm. Note: The data are represented as means ± SD. *p < 0.05, **p < 0.01, ***p < 0.001.

**Figure 7:**
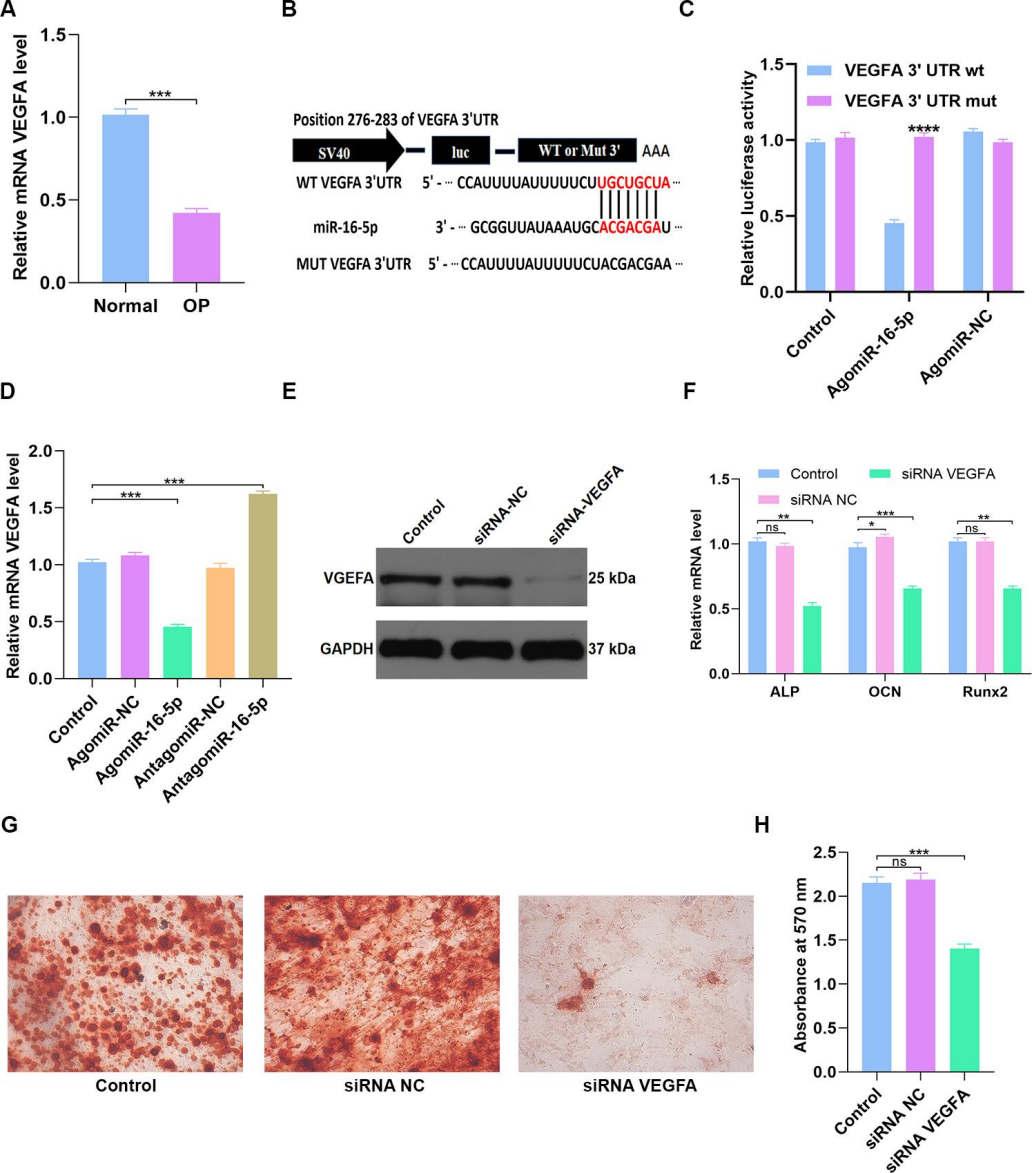
**miR-16-5p directly targets VEGFA.** (**A**) QRT-PCR analysis of VEGFA levels in postmenopausal patients with or without osteoporosis (n=10 per group). (**B**, **C**) Dual luciferase reporter assay results show firefly luciferase activity relative to Renilla luciferase activity in hMSCs transfected with dual luciferase vectors containing VEGFA-WT-3’UTR or VEGFA-MUT-3’UTR and miR-16-5p agonist (agomiR-16-5p) or negative control (agomiR-NC). (**D**) QRT-PCR analysis shows relative VEGFA mRNA levels in control hMSCs and hMSCs transfected with agomiR-NC, agomiR-16-5p, antagomiR-NC, and antagomiR-16-5p. (**E**) Western blotting analysis shows relative VEGFA protein levels expression in hMSCs transfected with siRNA-NC, and siRNA-VEGFA. (**F**) QRT-PCR analysis shows relative expression of osteogenic marker genes, ALP, OCN and RUNX2, in hMSCs transfected with siRNA-NC, and siRNA-VEGFA. (**G**, **H**) Alizarin red staining shows calcium deposition in hMSCs transfected with siRNA-NC, and siRNA-VEGFA for 21 days. Scale bar = 10 mm. The data are means ± SD. *p < 0.05, **p < 0.01, ***p < 0.001, ****p < 0.0001.

QRT-PCR analyses showed that miR-16-5p levels were significantly higher in the post-menopausal osteoporosis patients compared to postmenopausal women without osteoporosis (n=10 each; Figure 6A). Furthermore, we analyzed miR-16-5p and VEGFA levels in control hMSCs and hMSCs transfected with agomiR-NC, agomiR-16-5p, antagomiR-NC, and antagomiR-16-5p. The miR-16-5p levels inversely correlated with VEGFA protein expression compared to the corresponding controls (Figure 6B and Figure 7D). Moreover, the expression of osteogenic genes, ALP, OCN, and RUNX2 were significantly increased in the antagomiR-16-5p group, but significantly reduced in the agomiR-16-5p group (Figure 6C). This suggests that miR-16-5p suppresses osteogenesis. Furthermore, alizarin red staining showed increased calcium deposition in the antagomiR-16-5p group compared to the corresponding controls (Figure 6D–6E). This suggests that miR-16-5p expression decreases bone density.

### MiR-16-5p directly targets VEGFA

QRT-PCR analysis showed that VEGFA mRNA levels were significantly reduced in osteoporosis patients compared to healthy subjects (n=10 per group; p < 0.001; Figure 7A). This suggests that VEGFA levels correlate with osteoporosis progression. Next, we performed the dual luciferase reporter assay to confirm if miR-16-5p specifically binds to the 3’UTR of VEGFA mRNA. The results show high luciferase activity in the miR-16-5p plus Mutant type (Mut)-3’UTR-containing luciferase plasmid group, but, luciferase activity is significantly reduced in the miR-16-5p plus Wild type (WT)-3’UTR-containing luciferase plasmid group (Figure 7B–7C). These results demonstrate that miR-16-5p specifically targets VEGFA. Next, we analyzed control hMSCs and hMSCs transfected with agomiR-NC, agomiR-16-5p, antagomiR-NC, and antagomiR-16-5p. QRT-PCR analysis shows that VEGFA mRNA levels were significantly reduced in the agomiR-16-5p group and significantly increased in the antagomiR-16-5p group (p < 0.001; Figure 7D). These results confirm that miR-16-5p promotes osteoporosis by suppressing VEGFA expression.

Next, we transfected hMSCs with empty vector control or VEGFA-specific siRNAs (siRNA-NC or siRNA-VEGFA). Western blot analysis showed that VEGFA protein expression was significantly reduced in the siRNA-VEGFA group compared to the siRNA-NC group (Figure 7E). QRT-PCR analysis showed that the expression of osteogenic genes, ALP, OCN and RUNX2, was significantly reduced in the siRNA-VEGFA group compared to the siRNA-NC group (Figure 7F). This suggests that VEGFA promotes osteogenesis. Moreover, Alizarin red staining showed significantly reduced calcium deposition in the siRNA-VEGFA group compared to the siRNA-NC group (p < 0.001 Figure 7G–7H). This further suggests that VEGFA positively regulates bone density by promoting osteogenesis.

## DISCUSSION

In this study, we compared gene expression microarray data from 20 osteoporosis patients and 20 healthy subjects, and identified 638 DEGs, including 371 up-regulated genes and 267 down-regulated genes. We constructed a PPI network showing functional interactions between the DEGs and identified 10 hub genes, namely, *TP53, RPS27A, VEGFA, MAPK8, CDC42, CREBBP, SIRT1, RPL35A, RPL30,* and *SNRPG*, using CytoScaPe plugin of Cytoscape software.

The top three hub genes are *TP53, RPS27A, and VEGFA*. Jia et al. reported that pri-miR-34b/c rs4938723 and TP53 Arg72Pro polymorphisms contribute to the risk of osteoporosis [31]. Xie et al. reported that hMSCs from osteoporosis patients show significantly higher TP53 expression; moreover, TP53, SP1 and CTNNB1 transcription factors regulate most of the upregulated DEGs [32]. Felthaus et al. demonstrated that SP1 and TP53 regulate osteogenic differentiation in both dental follicle cells (DFCs) and stem cells from human exfoliated deciduous teeth [33]. The tumor suppressor p53 binds to OSX and prevents its binding to DLX5, thereby repressing osteoblast differentiation via deregulation of the osteogenic transcriptional network [34]. Several studies show that VEGFA plays an important role in osteoporosis [35, 36]. VEGFA is a pro-angiogenic factor that is upregulated in response to uniaxial cyclic tensile strain in human adipose-derived stem cells (hASCs) and hMSCs from osteoporotic donors [37]. In this study, we demonstrate that VEGFA is one of the top hub genes in the PPI network.

Hsa-miR-16-5p is the mature miRNA generated from precursor miRNAs such as miR-16-1, miR-16-2 [38]. Duan et al. reported that osteogenic differentiation is reduced by miR-16-2 upregulation and enhanced by downregulation of miR-16-2 [39]. Castaño et al. showed that antagomiR-16 increased the expression of a key osteogenic transcription factor RunX2 and the levels of OCN, an osteogenic biomarker [40]. Wang et al. showed that Hsa-miR-16 was differentially expressed in the sera of patients with osteogenesis imperfecta, a genetic bone disease [41]. In this study, we used TargetScanHuman and DIANA software and identified hsa-miR-16-5p as a potential miRNA that targets VEGFA mRNA. We confirmed this observation using dual luciferase reporter assays. Furthermore, we showed that miR-16-5p levels were upregulated and VEGFA levels were downregulated in osteoporosis patients. Moreover, alizarin red staining of hMSCs showed that high miR-16-5p expression reduced bone mineralization whereas miR-16-5p knockdown increased bone density.

Our study has a few limitations. First, we did not perform *in vivo* experiments to determine the role of miR-16-5p/VEGFA in osteoporosis. Secondly, we did not analyze other subtypes of osteoporosis in this study. Therefore, our results are applicable only to postmenopausal osteoporosis, which is the most common clinical subtype of osteoporosis. Further investigations are necessary to identify molecular mechanisms involved in other subtypes of osteoporosis. Moreover, future studies need to investigate the role of other miRNAs that regulate osteoporosis in order to gain a comprehensive understanding of the miRNA-mRNA axis that regulates osteoporosis.

In conclusion our study shows that miR-16-5p suppresses osteogenic differentiation by down-regulating VEGFA expression. Therefore, miR-16-5p/VEGFA axis is a potential therapeutic target for postmenopause-related osteoporosis.

## MATERIALS AND METHODS

### Data search and identification of deferentially expressed genes (DEGs)

The RNA microarray data of 20 postmenopausal women with osteoporosis and 20 healthy postmenopausal women in the GSE56815 dataset was retrieved from NCBI Gene Expression Omnibus (GEO) database. The raw gene expression data was log2 transformed and differentially expressed genes were identified using the GEO2R in-built function with a default setting (https://www.ncbi.nlm.nih.gov/geo/geo2r/). DEGs with a *P* < 0.05 were considered as statistically significant. A heatmap was constructed from the log2 mRNA expression data using the pheatmap R package.

### PPI network of the DEGs

We imported the DEGs into the Search Tool for the Retrieval of Interacting Genes (STRING) database [42] and identified protein-protein interactions with a combined score of >0.5. PPI networks were constructed using the Cytoscape software (version 3.7.2) [43]. Molecular Complex Detection (MCODE) plugin from Cytoscape was used to screen modules of the PPI network, and those with MCODE score ≥ 5 and number of nodes >5 were selected. CentiScaPe 2.2 plugin in Cytoscape was used to calculate the degree, betweenness, and closeness centralities of nodes in the PPI network. Node degree is a measure that represents the number of connections associated with a specific node in the network. Closeness centrality defines how close a node is to all other nodes in the network. Betweenness centrality is the number of times a node acts as bridge along the shortest path between two other nodes.

### GO and KEGG pathway enrichment analysis

The function and pathway enrichment analyses of the candidate genes were performed using the DAVID. GO annotation was performed to identify top 3 enriched GO terms associated with biological processes, molecular functions and cellular components. Moreover, the top enriched KEGG pathways involved in osteoporosis were also analyzed using DAVID.

### Circular visualization

The top ten genes with the highest degrees (hub genes) were visualized using the ggplot2 software [44]. GO plot was used to visualize the results of hub gene enrichment analysis [45]. Circular Visualization in R [46] was used to visualize chromosomal positions in a circular representation and the degree connectivity of the top 100 genes.

### Cell culture and transfection

The hMSCs were obtained from the Huazhong University of Science and Technology, Wuhan, China and grown in a specific media designed for human mesenchymal stem cells (#MUXMA-90011, Cyagen, Suzhou, China) at 37°C in a 5% CO_2_ incubator. They were maintained for a maximum of 3 passages for experiments. The agomiR-16-5p, antagomiR-16-5p, agomiR-NC, antagomiR-NC, si-NC and si-VEGFA constructs were obtained from GenePharma (Shanghai). Cell transfection experiments were performed using Lipofectamine 3000 (ThermoFisher Scientific) according to manufacturer’s instructions using miRNA constructs at 200 mM and siRNA constructs at 50 mM.

### Blood collection

From May 2016 to June 2018, peripheral blood samples from patients in Shanghai Tongji Hospital (10 healthy volunteers, 10 postmenopausal osteoporosis patients) were collected for gene expression analysis. The patient studies were approved by the Committees of Clinical Ethics in the Tongji Hospital (Tongji University of Medicine, Shanghai, China), and informed consent was obtained from all participants.

### QRT-PCR analysis

Total RNA was extracted using Trizol (#15596018, Invitrogen, USA) and reverse transcribed using the Verso^TM^ cDNA Synthesis Kit (#AB-1054/A, ThermoFisher Scientific) according to the manufacturer’s protocol. The exosomal miRNAs were isolated using the SeraMir Exosome RNA purification Kit (System Biosciences, USA), and reverse transcribed and quantified using the TaqMan microRNA assay kit (Applied Biosystems, USA) for cDNA synthesis and qRT-PCR. The qRT-PCR reactions were performed using the Thermal Cycler C-1000 Touch system (#10021377, Bio-Rad CFX Manager, USA). U6 was used as a control for miRNA quantification whereas GAPDH was used as the internal control to quantify mRNA. Osteogenesis was estimated by analyzing the expression of osteogenic marker genes, ALP, RUNX2 and OCN by qRT-PCR analysis. The data was expressed as fold change relative to the appropriate controls. All the primers used in this study are listed in Table 3.

**Table 3:** QRT-PCR primers used in the study.

**Gene name**	**Primer sequence**
hsa - miR-16-5p - Forward	TGGGGTAGCAGCACGTAAA
hsa - miR-16-5p - Reverse	CTCAACTGGTGTCGTGGAGTC
hsa-U6-Forward	CTCGCTTCGGCAGCACA
hsa-U6-Reverse	AACGCTTCACGAATTTGCGT
hsa-VEGFA-Forward	AGGGCAGAATCATCACGAAGT
hsa-VEGFA-Reverse	AGGGTCTCGATTGGATGGCA
hsa-ALP - Forward	ACCACCACGAGAGTGAACCA
hsa-ALP - Reverse	CGTTGTCTGAGTACCAGTCCC
hsa-OCN - Forward	CAAAGGTGCAGCCTTTGTGTC
hsa-OCN - Reverse	TCACAGTCCGGATTGAGCTCA
hsa-Runx2 - Forward	TGGTTACTGTCATGGCGGGTA
hsa-Runx2 - Reverse	TCTCAGATCGTTGAACCTTGCTA
hsa-GAPDH - Forward	CCGTTGAATTTGCCGTGA
hsa-GAPDH - Reverse	TGATGACCCTTTTGGCTCCC

### Western blotting

Total protein samples were prepared from cells and callus samples using Protein Lysis buffer (#AS1004, Aspen, South Africa) containing 1% protease inhibitor (#AS1008, Aspen). Equal amounts of protein samples were separated on SDS-PAGE and transferred onto nitrocellulose membranes (#IPVH00010, Millipore, USA). Then, the membranes were blocked by incubation with 5% nonfat milk for 1 h followed by overnight incubation at 4°C with primary antibodies against VEGFA (1:500, Sigma, USA, #HPA069116) and GAPDH (1:10,000, Abcam, USA, #ab37168). Then, the blots were incubated with HRP-conjugated secondary antibody (#AS1058, Aspen). The blots were developed using enhanced chemiluminescence detection system and the expression of VEGFA relative to GAPDH was determined for all samples. Each experiment was repeated three times.

### Alizarin red staining

The hMSCs were grown in 6-well plates in specific media containing 100 nM dexamethasone, 50 mM ascorbic acid, and 10 mM b-glycerophosphate to promote osteogenesis (#HUXMA-90021, Cyagen, USA). Then, the cells were washed twice with 1X PBS and fixed in 10% formalin for 15 minutes. Subsequently, the cells were stained with 1 mL 0.5% alizarin red staining solution at room temperature for 15 minutes. After rinsing the cells with distilled water for 5 minutes, the cells were mounted on slides and analyzed for red mineralized nodules using the charge-coupled device microscope. The absorbance was measured at 570 nm. The experiments were repeated in triplicate.

### Luciferase reporter assays

The hMSCs were grown in 24-well plates (2.5×10^5^ cells/well) and transfected with dual-luciferase vectors containing VEGFA-WT-3’UTR and VEGFA-MUT-3’UTR plus miR-16-5p agonist (agomiR-16-5p) or negative control (agomiR-NC). A Quik Change Site-Directed Mutagenesis Kit (Strata gene) was used to mutate the miR-16-5p binding-region in the VEGFA-3’UTR. The dual luciferase reporter kit (Promega) was used to perform the luciferase assay according to the manufacturer’s instructions. A luminometer (Glomax, Promega) was used to quantify luminescence from the firefly luciferase and control Renilla luciferase constructs in each group. The values of firefly luciferase activity were normalized to the corresponding Renilla signal.

### Statistical analysis

**Figure 8:**
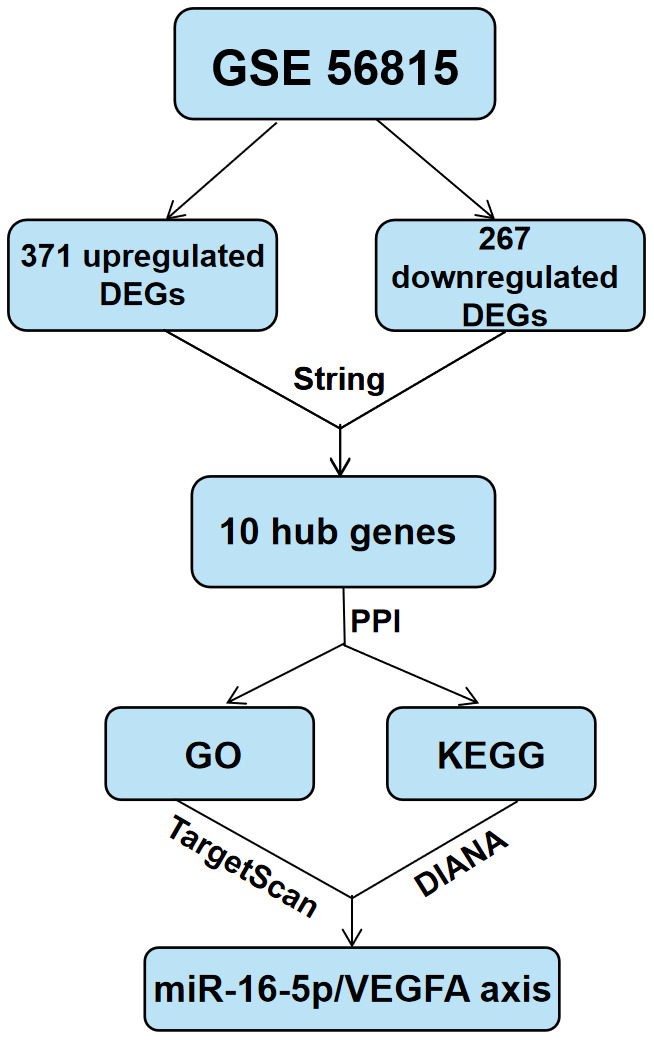
**Schematic representation of the experimental strategy in the present study.**

The data are presented as means ± SD. Binary groups were compared using Student's t-test,, whereas, multiple groups (more than two) were compared using one-way ANOVA with Tukey post hoc test. The statistical analyses were conducted using the Graphpad Prism 8.0 software (Graphpad software, San Diego, CA, USA). P<0.05 was considered statistically significant. The schema of the approach process in this study is shown in Figure 8.

## Supplementary Material

Supplementary Figure 1
