# Supplementary material for: MiR-16-5p regulates postmenopausal osteoporosis by directly targeting VEGFA
Source: Aging (Albany NY). 2020 May 19;12(10):9500–14. doi: 10.18632/aging.103223 (PMC7288956; doi:10.18632/aging.103223)
Supplement: Supplementary Figure 1 [file aging-12-103223-s001..pdf]

SUPPLEMENTARY FIGURE

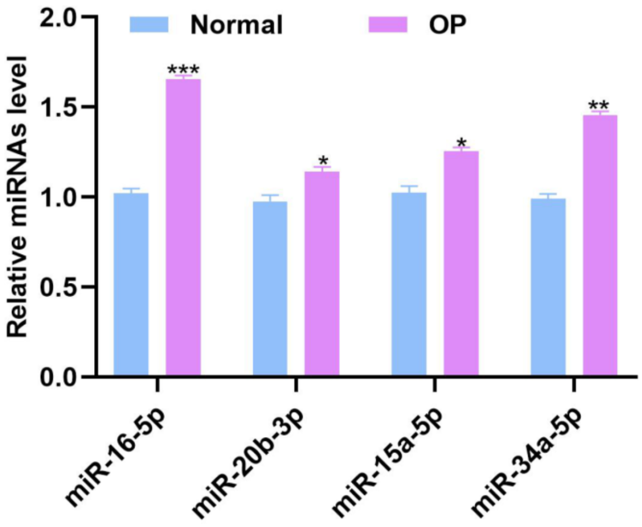

Supplementary Figure 1. The expression of the related miRNAs between the non-OP patients (n=10) and the OP patients (n=10) were measured by qRT-PCR analysis.
